# Effect of hypoglycemic events on cognitive function in individuals with type 2 diabetes mellitus: a dose–response meta-analysis

**DOI:** 10.3389/fneur.2024.1394499

**Published:** 2024-08-13

**Authors:** Min Ye, Qiqi Yang, Lele Zhang, Hudie Song, Qin Fu, Jun Qian, Hongyu Xie, Aihong Yuan

**Affiliations:** ^1^First School of Clinical Medicine, Anhui University of Chinese Medicine, Hefei, Anhui, China; ^2^Acupuncture and Rehabilitation Department, The First Affiliated Hospital of Anhui University of Chinese Medicine, Hefei, Anhui, China

**Keywords:** type 2 diabetes mellitus, cognitive dysfunction, hypoglycemia, dose–response analysis, meta-analysis

## Abstract

**Background:**

Type 2 diabetes mellitus (T2DM) is widely acknowledged as a vital warning sign contributing to cognitive dysfunction. However, there is still a lack of consensus on whether hypoglycemic events resulting from poor glycemic control increase the risk of cognitive dysfunction in people with diabetes, and the potential dose–response correlation between hypoglycemic events and cognitive dysfunction remains unexplored. The primary objective of the current study was to assess the contribution of hypoglycemic events to cognitive dysfunction in T2DM patients and the dose–response correlation between the two.

**Methods:**

A comprehensive search of nine major databases was executed from inception to May 2023. We screened all observational studies examining the connection between hypoglycemia and cognitive dysfunction. The DerSimonian-Laird method was used to compute the combined risk ratio (RR) and its 95% confidence interval (CI). Additionally, dose–response analysis was employed to investigate the correlation between the frequency of hypoglycemia and the likelihood of cognitive dysfunction.

**Results:**

A total of 30 studies of different levels in 17 articles with 3,961,352 participants were included in this review. The pooled RR for the connection of hypoglycemia and the likelihood of cognitive dysfunction was 1.47 (95% CI: 1.35–1.60). Subgroup analyses showed that the pooled RR for the likelihood of cognitive dysfunction was 1.20 (95% CI: 1.11–1.31) for one episode of hypoglycemia, 1.41 (95% CI: 1.05–1.88) for two episodes of hypoglycemia, and 1.62 (95% CI: 1.20–2.91) for three or more episodes of hypoglycemia. Dose–response analysis showed a linear dose–response relationship between hypoglycemia and the likelihood of cognitive dysfunction (exp (*b*) = 1.178694, *z* = 7.12, *p* < 0.001).

**Conclusion:**

Our investigations demonstrated a 47% heightened likelihood of cognitive dysfunction in individuals with hypoglycemia compared to those without. Furthermore, the likelihood of cognitive dysfunction climbed by 17.87% for every subsequent episode of hypoglycemia. Therefore, long-term monitoring of blood glucose, periodic screening of cognitive function, and moderate health education should be encouraged, which will be beneficial for people with diabetes to prevent hypoglycemic events and cognitive dysfunction.

**Systematic review registration:**

https://www.crd.york.ac.uk/PROSPERO/, CRD42023432352.

## 1 Introduction

Diabetes mellitus has emerged as a widespread metabolic condition accompanied by hyperglycemia (1). It is estimated that the global prevalence of diabetes among individuals aged 20–79 years was 10.5% in 2021, and this number is projected to rise to 12.2% by 2045, following predictions by the International Diabetes Federation (2). The most prevalent subtype of diabetes mellitus is type 2 diabetes mellitus (T2DM), which makes up around 90% of all cases (3). Notably, individuals with T2DM have a significantly elevated likelihood of cognitive disorders, highlighting cognitive dysfunction as a crucial complication of diabetes (4). Consequently, T2DM has been highlighted as an independent risk factor for cognitive dysfunction when compared to individuals without diabetes (5–7).

It has become widely accepted that T2DM and cognitive dysfunction are progressive diseases closely related to age, which have a high prevalence in elderly people and those of middle age. With the development of a globally aging society and the extension of life expectancy of people with T2DM worldwide, T2DM complicated with cognitive dysfunction has become a great pressure and challenge for the development of society and economy currently. However, up to now, there is still no effective cure for cognitive dysfunction worldwide. Recently, two large-scale clinical trials on drug prevention and treatment of Alzheimer’s disease ended in failure, and one phase III clinical trial had to be terminated because of no efficacy (8, 9). With the failure of pharmacotherapy for cognitive dysfunction and the related side-effects becoming increasingly prominent, it is particularly important to explore effective prevention measures for cognitive dysfunction, especially for diabetes-related cognitive dysfunction, whose prevention strategies still need to be improved.

Glycemic control is the core goal of the treatment strategy for people with T2DM, and it is also the most commonly used prevention and treatment method in clinical practice. As a result, hypoglycemic events and consequent cognitive decline caused by pharmacotherapies for glycemic control are common in clinical practice. The amygdala, hippocampus and prefrontal cortex (PFC) are the core brain regions for cognitive formation and maintenance (10), and glucocorticoid receptors have been found to be widely distributed in these brain regions (11). Interestingly, glucocorticoid function mediated by glucocorticoid receptors is also involved in the regulation of blood glucose while being involved in cognitive maintenance (12). Some mechanistic studies suggest that hypoglycemic events may affect cognition by affecting neuroinflammation, oxidative stress, mitochondrial dynamics, and energy metabolism in these brain regions (13–16). However, the current clinical evidence on whether hypoglycemic events enhance the likelihood of cognitive deficit in people with T2DM remains controversial. Several studies from different countries have maintained that hypoglycemic events affect cognitive function in people with T2DM and increase the risk of progression to cognitive deficit (17–20). Nevertheless, two other large cohort studies involving multiple countries have maintained that hypoglycemic events do not affect cognitive function, nor do they increase the possibility of cognitive decline progressing to cognitive dysfunction (21–23). These two almost contradictory conclusions have caused serious confusion and great challenges in the development of hypoglycemic regimens and the implementation of intensive glucose control. Thereby, it is a pressing need to identify the exact connection between hypoglycemic events and the likelihood of cognitive dysfunction, and to provide evidence-based recommendations and guidance for structuring glycemic control plans and cognitive dysfunction prevention strategies.

In 2022, Maria Dolores Gomez-Guijarro’s team (24) conducted a systematic review and meta-analysis of studies on the connection between severe hypoglycemic incidents and dementia in individuals concerning T2DM. However, they only included 7 eligible literature in the final evaluation due to the dearth of pertinent studies at the time, and the significant heterogeneity among the literature found. Simultaneously, a substantial portion of the literature indicates a moderate susceptibility to bias, as acknowledged by the authors themselves who caution against over-interpretation of their findings. Consequently, there is an absence of dependable evidence-based outcomes to substantiate the impact of hypoglycemic events on cognitive competence in individuals concerning T2DM. To elucidate the correlation between occurrences of hypoglycemia and cognitive dysfunction, this study incorporated the most recent investigations, revised the systematic review and meta-analysis, and additionally investigated the dose–response connection between hypoglycemic events and cognitive dysfunction.

## 2 Methods

This research is designed to be a dose–response meta-analysis. Before undertaking this research, we duly registered the study protocol with the PROSPERO database. The study protocol has been published online (25), but due to the limited amount of literature, only part of the original protocol has been completed in this study. The execution of this study adhered to the procedure outlined in the Cochrane Handbook for Systematic Reviews of Interventions (26), and the subsequent reporting followed the Preferred Reporting Items for Systematic Reviews and Meta-Analysis Statement (PRISMA) and the guideline of Meta-analyses of Observational Studies in Epidemiology (MOOSE) (27, 28).

### 2.1 Search strategy

A thorough search was carried out across various databases including PubMed, Web of Science, CBM, Cochrane Library, Embase, CNKI, Wan Fang, Wei Pu, and Du Xiu, covering the period from inception to May 2023. The search utilized Medical Subject Headings (MeSH) and free terms, such as ‘diabetes mellitus, type 2’, ‘noninsulin’, ‘noninsulin-dependent diabetes mellitus’, ‘type 2 diabetes’, ‘type 2 diabetes mellitus’, ‘type 2 diabetic’, ‘T2DM’, ‘DM’, ‘cognitive dysfunction’, ‘cognition disorders’, ‘cognitive disorder’, ‘dementia’, ‘cognitive decline’, ‘cognition disorder’, ‘cognitive deficit’, ‘cognitive impairment’, ‘executive function’, ‘cognitive function’, ‘memory’, ‘risk factor’, ‘predicted’, ‘predictor’, ‘risk’, ‘relat’, ‘associat’, ‘factor’, ‘reason’, ‘correlated’, ‘predictor’, ‘influen’, ‘inciden’, for comprehensive coverage. Furthermore, reference lists of relevant articles were manually searched and records from relevant trial registries were retrieved. The search strategy was finally formulated based on the results of repeated pre-search to include relevant studies as comprehensively as possible. The search strategy was shown in Supplementary material 1.

### 2.2 Inclusion criteria

We contained studies meeting all the given requirements.

#### 2.2.1 Type of studies

All observational studies published in Chinese or English were included.

#### 2.2.2 Type of participants

Individuals who have been medically diagnosed with type 2 diabetes and have been at least 18 years old are eligible to participate. Prior to enrollment and at the time of enrollment, every participant had normal cognitive function. The study has no restrictions on sex, race, duration of diabetes, or severity of diabetes among the participants.

#### 2.2.3 Criteria for the assessment of hypoglycemia

The plasma glucose level was less than 3.9 mmol per liter (29), and the patient showed symptoms such as palpitation and dizziness.

#### 2.2.4 Contents of studies

Studies that explored the connection between hypoglycemia and the likelihood of cognitive dysfunction were included. The included studies had to provide data on the connection between the two, such as odds ratio [OR], risk ratio [RR], hazard ratio [HR], and 95% confidence intervals.

### 2.3 Exclusion criteria

Studies were disqualified if they fulfilled any of the specified criteria.

1. Duplicate publication of the same study (studies that have more detailed and credible results, are more recent, or have a larger sample size will be selected);
2. Studies for which full text or relevant data are not available;
3. Studies with insufficient methodological details or poor quality (e.g., high risk of bias, inadequate statistical analysis);
4. Studies with comorbid conditions that could independently affect cognitive function (e.g., neurodegenerative diseases other than diabetes-related cognitive impairment);
5. Studies where the dose of hypoglycemic events is not clearly defined;
6. Studies not published in English or Chinese;
7. Studies with a duration that is too short to capture meaningful cognitive changes.

### 2.4 Study selection

The literature was managed using NoteExpress software. Two reviewers (Hongyu Xie and Qiqi Yang) screened the literature independently. Duplicate literature was eliminated first. Then, the literature that did not fit the research theme was eliminated through the title and abstract. After that, the extra literature was carefully reviewed in its full text and assessed against the inclusion and exclusion criteria. In case of disagreement, a compromise was established after discussing with an additional reader (Lele Zhang).

### 2.5 Data extraction

The information that followed was obtained independently by two researchers (Lele Zhang and Qin Fu).

1. General information of studies: the primary author, period of publication, country and region, study design type, and duration of follow-up;
2. Participant characteristics: average age, sample size;
3. Disease characteristics: diagnostic criteria for hypoglycemia, type of diabetes mellitus, frequency of hypoglycemic events, and diagnostic criteria for cognitive dysfunction.

### 2.6 Risk of bias assessment

The involved experiments’ levels of quality and publication bias were evaluated using the Newcastle-Ottawa Scale (NOS). The case–control study’s primary evaluation criteria focused on subject selection (4 points), group comparability (2 points), and exposure factor measurement (3 points). Cohort study grading criteria included subject selection (4 points), group comparability (2 points), and outcome measurement (3 points). The total score for both evaluations was 9 points. The quality of studies was judged and categorized as ‘High’, ‘Medium’, or ‘Low’ based on the scoring criteria, with scores above six indicating high quality, scores of five indicating medium quality, and scores below five indicating low quality (30). Two researchers (Jun Qian and Hudie Song) separately conducted and cross-checked the risk of bias assessment, while a third researcher (Lele Zhang) worked to resolve any discrepancies.

### 2.7 Statistical analysis

The DerSimonian-Laird method was employed to generate the pooled RR and its 95% confidence interval (CI) (31). When RR was not provided in the literature, specific formulas were used to convert OR and HR to RR (32–34). To gage the heterogeneity of the considered literature, the Chi-square test and *I*^2^ statistic were utilized. When the value of *I*^2^ was less than 30%, the fixed-effects model was employed; Otherwise, the random-effects model was adopted. The origins of heterogeneity were investigated and the stability of the combined findings was assessed utilizing sensitivity and subgroup analyses (35). The relationship between the frequency of hypoglycemia events and the likelihood of cognitive dysfunction was examined using dose–response analysis. To determine whether the results were influenced by participant age, study type, nation and area, and follow-up period, meta-regression was employed. Observation of funnel plots and Egger’s regression asymmetry test were both employed for assessing publication bias (36). The STATA SE program, version 15 (StataCorp), was used to carry out the aforementioned statistical analysis.

## 3 Results

### 3.1 Study inclusion and characteristics

**Figure 1:**
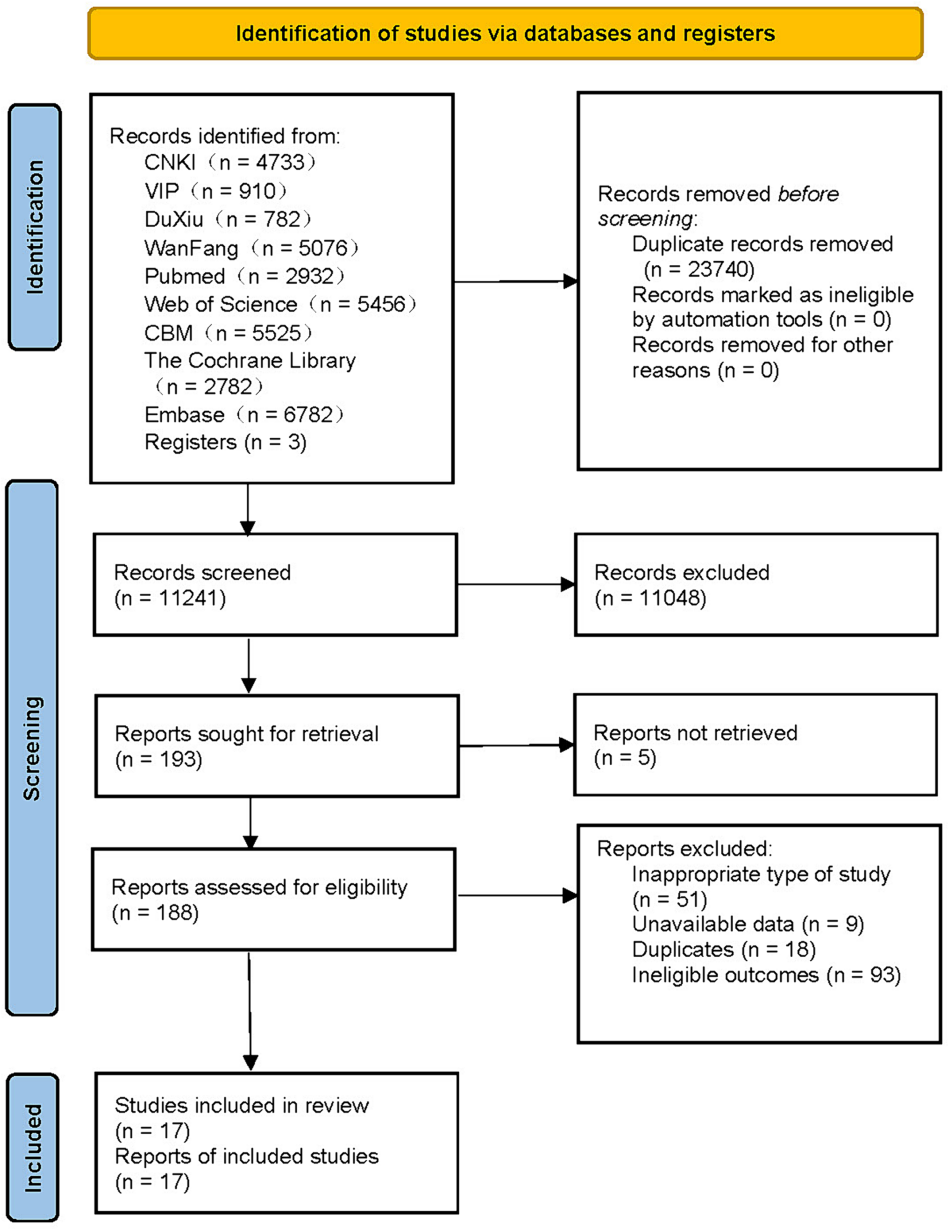
Flow chart of literature screening.

There were 34,981 relevant studies in total that had been found, and 17 of them satisfied the requirements to be considered in the meta-analysis (Figure 1) (17–21, 37–46). These investigations were conducted across a wide range of nations, including China, the United States, the United Kingdom, South Korea, Canada, and many others. Four studies were published in Chinese (37–40), and the remaining 13 were all published in English (17–21, 41–48). Among them, five studies were designed as case–control studies (37–40, 45), and the remaining 12 were cohort studies (17–21, 41–44, 46–48). Each study incorporated in this research was published within the timeframe of 2009–2023, encompassing a follow-up span ranging from 1 to 27 years. The sample sizes of these studies varied, ranging from 90 to 2,032,689 participants, resulting in a cumulative inclusion of 3,961,352 participants for this meta-analysis. Most of the participants were middle-aged or elderly individuals with T2DM, and three studies did not identify the participants’ specific type of diabetes (43, 44, 48).

For the diagnosis and quantification of hypoglycemic events, questionnaires, medical records, and medical insurance records were mostly used. Scale scores, medical records, and medical insurance records were primarily used for the identification and quantification of cognitive dysfunction. One of these investigations looked into the connection for cognitive dysfunction risk and hypoglycemia occurrences in a middle-aged cohort and an elderly cohort respectively (17). Six studies investigated the connections between different numbers of hypoglycemic incidents and the likelihood of cognitive dysfunction (19, 20, 41, 44, 46, 47). Our criteria were met by a total of 17 articles involving 30 pertinent investigations at various levels. Except for four studies that did not specify the adjustment for confounders (37–40), the remaining studies clearly showed the adjusted confounders. Detailed information is provided in Supplementary material 2.

### 3.2 Quality assessment of studies included

The findings of the NOS assessment revealed that the included papers had moderate or higher quality, indicating a low risk of publication bias and credible conclusions (Table 1).

**Table 1:** Quality assessment of studies included.

Author	Year	Selection	Comparability	Measurement of outcome/exposure factors	Score	Quality grades
Wajd Alkabbani	2023	4	2	3	9	High
Chung-Yi Li	2022	4	2	3	9	High
Eugene Han	2022	4	2	3	9	High
Zhang Jie	2021	3	0	2	5	Middle
YoungGun Kim	2020	4	2	3	9	High
Hou Xinghua	2019	3	0	2	5	Middle
Mao Hongling	2019	3	0	2	5	Middle
Shuling Liu	2019	4	1	3	8	High
Tali Cukierman-Yaffe	2019	4	2	3	9	High
Hemalkumar B	2017	4	2	3	9	High
Liu Chang	2016	3	1	2	6	High
Sang Ouk Chin	2016	4	2	2	8	High
Nisha Nigil Haroon	2015	4	2	3	9	High
Insa Feinkohl	2014	4	2	2	8	High
C-H Lin	2013	4	2	2	8	High
Kristine Yaffe	2013	4	2	3	9	High
Rachel A. Whitmer	2009	3	2	3	8	High

### 3.3 Meta-analysis and sensitivity analysis

**Figure 2:**
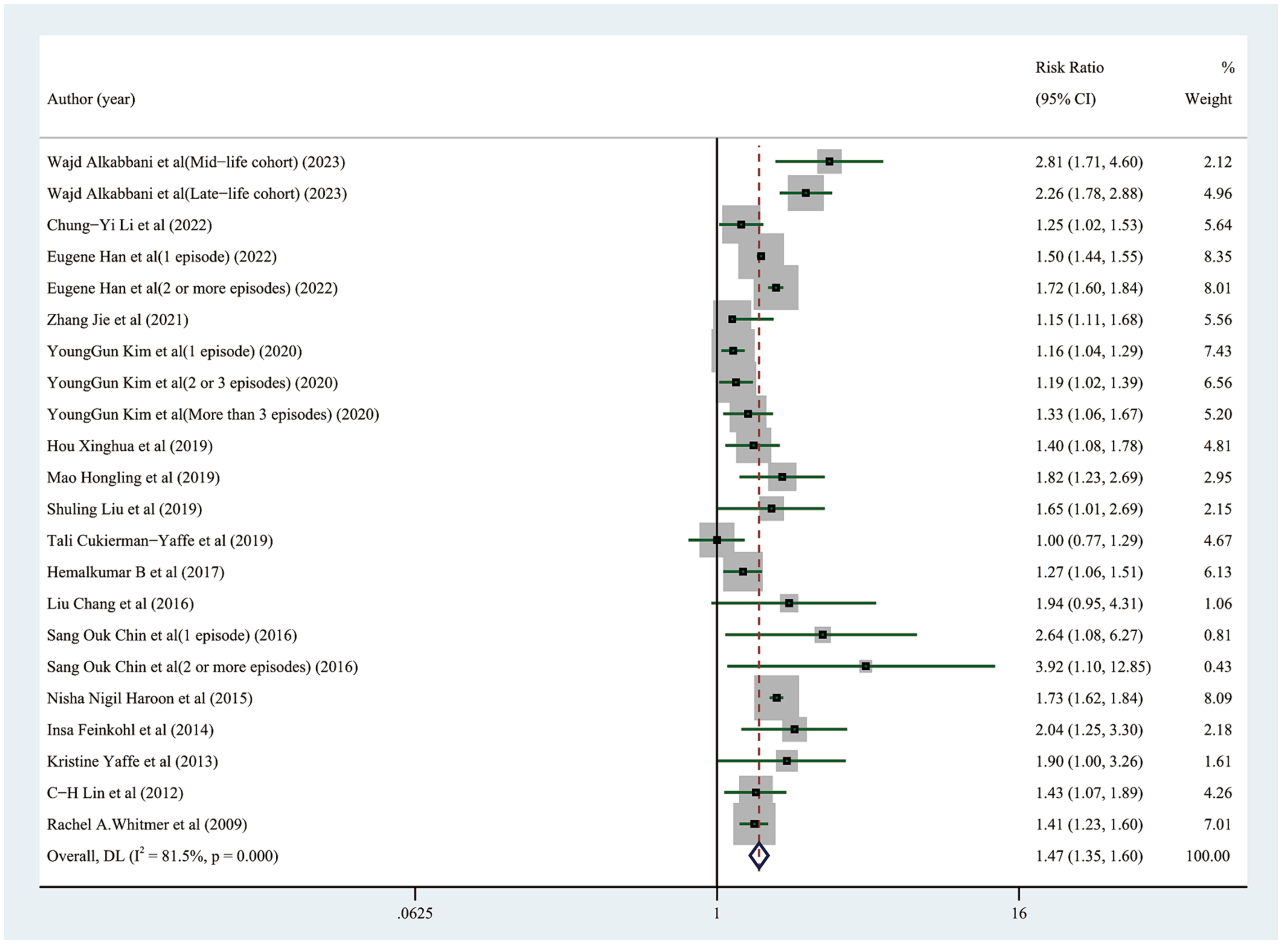
Forest plot showing the pooled risk ratio for the risk of cognitive impairment in diabetic participants with and without any hypoglycemic events.

Thirty studies of the 17 articles at different levels were screened and classified. Twenty-two studies examined the likelihood of cognitive dysfunction in diabetic participants who had or did not have any hypoglycemic events, and the pooled RR was 1.47 (95% CI: 1.35–1.60) (Figure 2). The heterogeneity among the 22 studies included in this study was significant (*I*^2^ = 81.5%, *p*<0.001), hence the sensitivity analysis was carried out by removing a single study one at a time to identify the source of heterogeneity. After excluding 6 studies by Wajd Alkabbani et al., the heterogeneity was significantly reduced (*I*^2^ = 46.4%, *p* = 0. 022), and 1.45 (95% CI: 1.33–1.58) for the pooled RR of the other 16 trials, indicating that hypoglycemic events still significantly increased the risk of cognitive dysfunction (Supplementary material 3). After further excluding 2 studies by Zhang Jie et al., the heterogeneity was further reduced (*I*^2^ = 18.1%, *p* = 0.257), and the cumulative RR of the rest 14 trials was 1.48 (95% CI: 1.43–1.53), demonstrating that there was still a substantial effect of hypoglycemia events on the chance of cognitive dysfunction (Supplementary material 4). In this sensitivity analysis, we found a substantial reduction in heterogeneity after the exclusion of studies with participants who were too old or too young, with too many episodes of hypoglycemia, or with particularly severe hypoglycemia, but these did not affect the overall trend of our pooled results, which makes our findings robust.

### 3.4 Dose–response analysis

**Figure 3:**
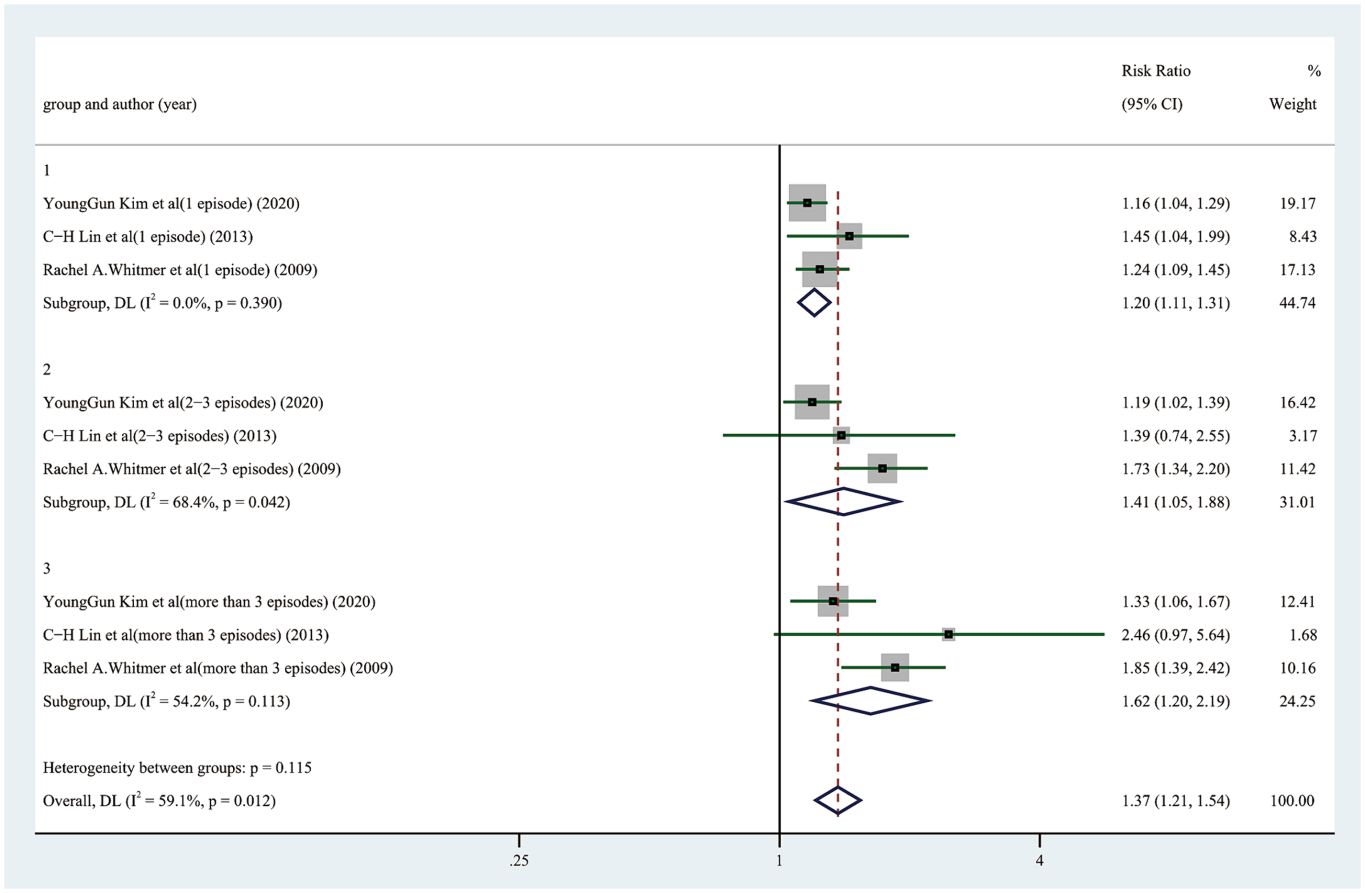
Forest plot showing the pooled risk ratio for the likelihood of cognitive dysfunction in diabetic participants with different episodes of hypoglycemia.

**Figure 4:**
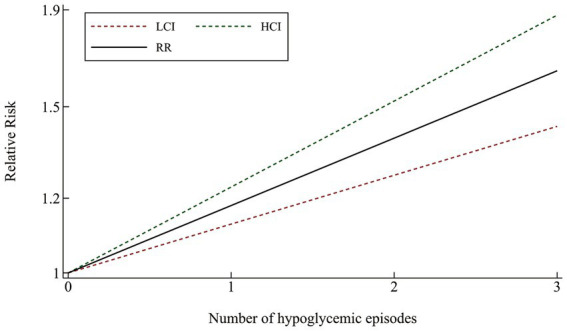
Linear dose–response connection between the number of hypoglycemia episodes and relative risk of cognitive impairment.

The likelihood of cognitive dysfunction rose as the frequency of hypoglycemia incidents increased as follows: (1) The pooled RR of one hypoglycemic event was 1.20 (95% CI: 1.11–1.31), and there was barely any heterogeneity among the trials (*I*^2^ = 0.0%, *p* = 0.390); (2) The pooled RR of two hypoglycemic events was 1.41 (95% CI: 1.05–1.88), and the heterogeneity among trials was considerable (*I*^2^ = 68.4%, *p* = 0. 042); The pooled RR for three or more episodes of hypoglycemia was 1.62 (95% CI: 1.20–2.19), and the heterogeneity among trials was moderate (*I*^2^ = 54.2%, *p* = 0.113) (Figure 3). A more thorough examination of the dose–response analysis revealed a linear dose–response association between the frequency of hypoglycemic events and the likelihood of cognitive dysfunction (exp (*b*) = 1.178694, *z* = 7.12, *p* < 0.001) (Figure 4).

### 3.5 Subgroup analysis and meta-regression analysis

**Figure 5:**
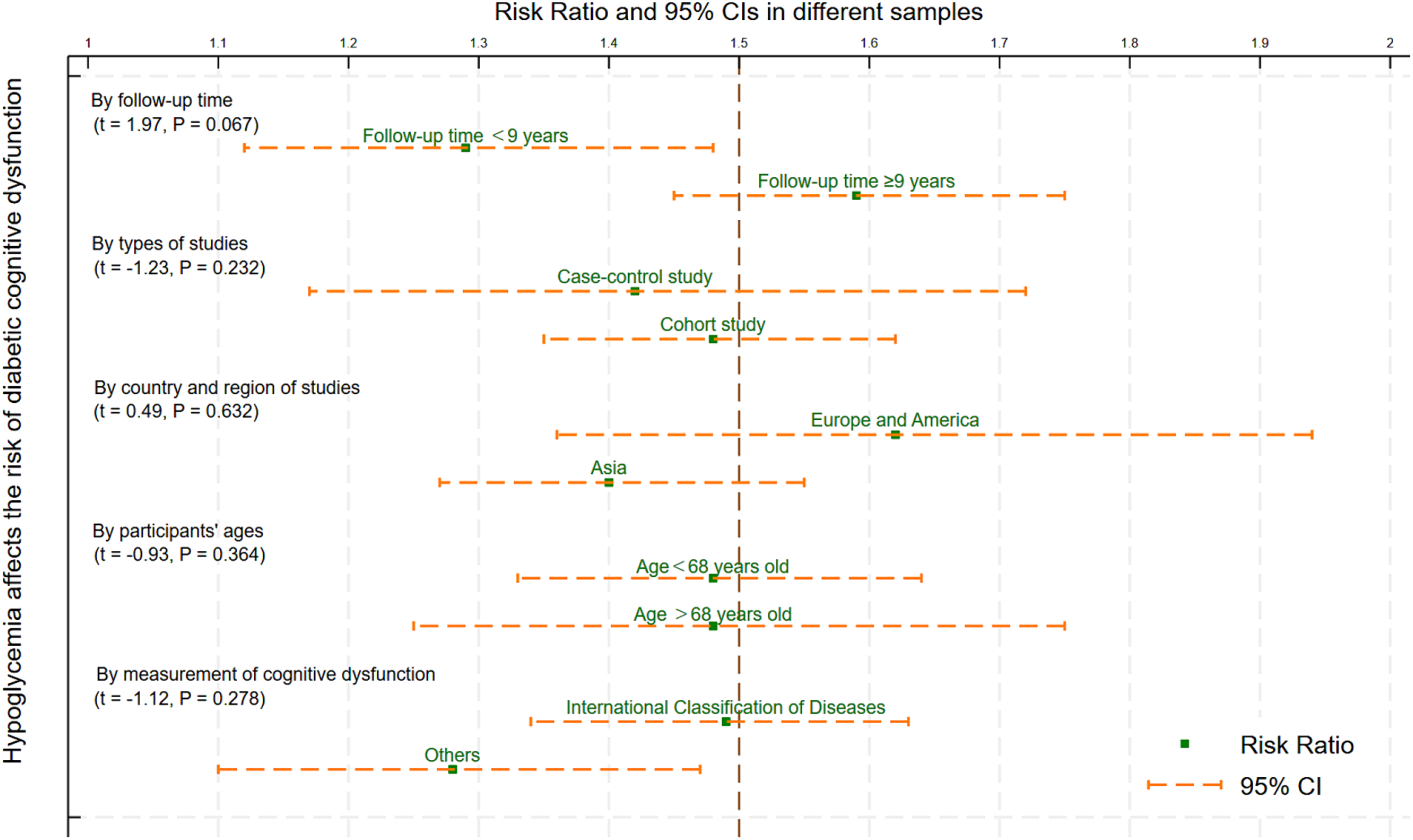
Meta-regression and subgroup analysis of the variables affecting hypoglycemia and cognitive impairment.

The median follow-up time (9 years), types of studies (case–control study and cohort study), country and region of studies (Asia, Europe and America), median age (68 years), the measurement of cognitive dysfunction (International Classification Diseases and others) were used for subgroup analysis and meta-regression. The findings highlighted that the follow-up time (*t* = 1.97, *p* = 0.067), types of studies (*t* = −1.23, *p* = 0.232), country and region of studies (*t* = 0.49, *p* = 0.632), participants’ ages (*t* = −0.93, *p* = 0.364), and the measurement of cognitive dysfunction (*t* = −1.12, *p* = 0.278) were not the source of heterogeneity and influencing factors of this review (Figure 5).

### 3.6 Publication bias

**Figure 6:**
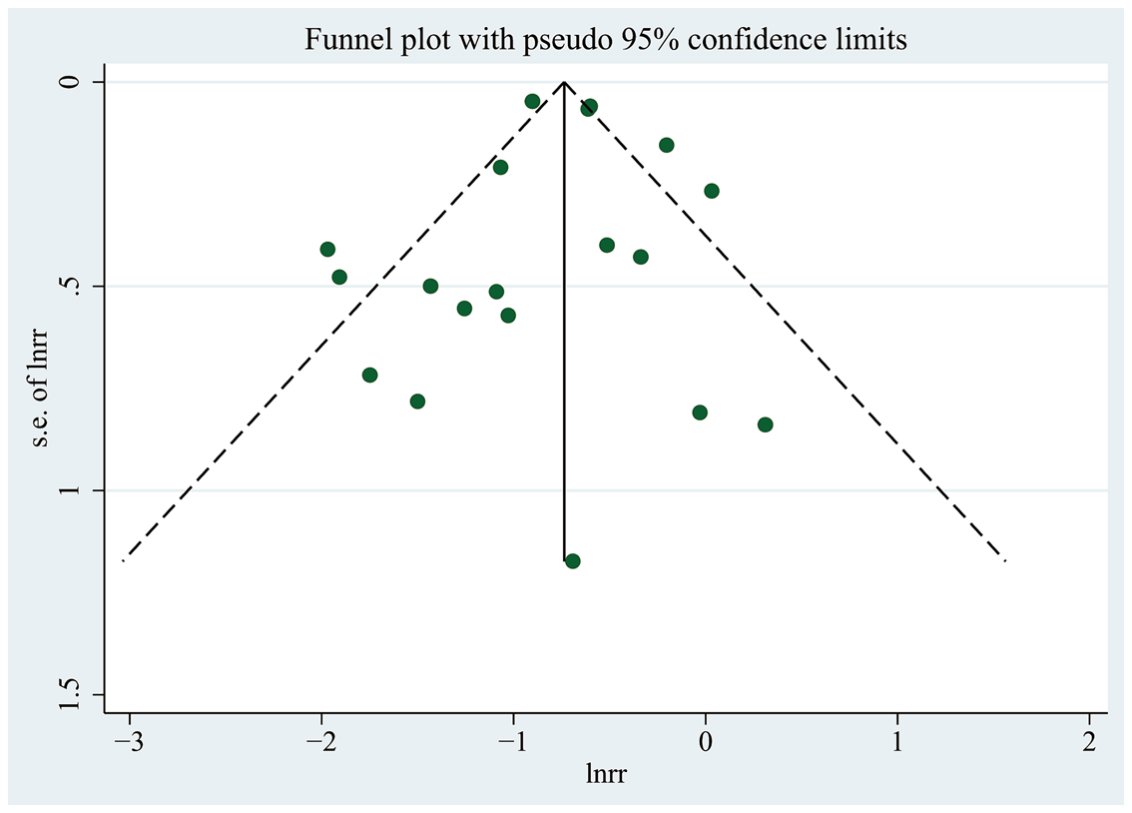
Funnel plot for the likelihood of cognitive dysfunction in diabetic participants with and without any hypoglycemic events.

Observation of funnel plots (Figure 6) and Egger’s regression asymmetry test did not reveal any indications of publication bias (*p* = 0.853).

## 4 Discussion

This study provides the latest summary of the connection between hypoglycemia and cognitive dysfunction risk in individuals with T2DM. Our findings showed that the likelihood of cognitive dysfunction was increased by 47% in those who experienced hypoglycemia compared with those who did not. Sensitivity analysis showed that after excluding any study or heterogeneous studies, the results of this study still maintained a momentous positive correlation, signifying their reliability. Furthermore, the frequency of hypoglycemia was found to be strongly linked to a higher risk of cognitive dysfunction, as indicated by subgroup and dose–response analysis. The meta-regression results indicated that participant age, follow-up duration, study type, country, and region little influenced the study’s conclusion. Additionally, no evidence of publication bias was found in this review. However, some small sample studies were included in this study, and in fact, they may have a certain publication bias on the overall research results.

Our findings confirmed that middle-aged and elderly individuals with T2DM face a significantly higher likelihood of cognitive dysfunction due to hypoglycemic events. This finding aligns with the research conducted by Maria Dolores Gomez - Guijarro’s group (24). To our excitement, this study, to our knowledge, is the first to demonstrate a Linear dose–response connection between hypoglycemic events and the likelihood of cognitive dysfunction, using both subgroup and dose–response analyses. Specifically, each additional episode of hypoglycemia would contribute to an approximately 17.87% increase in the likelihood of cognitive dysfunction (limited to this model). This finding suggests that there may be a cumulative clinical effect of brain damage caused by hypoglycemic events. Simply put, a greater frequency of hypoglycemic episodes increased the likelihood of cognitive dysfunction. Fortunately, most brain injuries caused by hypoglycemic events are reversible in the early stages. Therefore, timely detection of hypoglycemic events and correction of blood glucose levels can promote the recovery of brain function (49, 50) and avoid the cumulative damage of cognitive function caused by repeated hypoglycemic events. Strengthening health education about hypoglycemia events and providing continuous, effective, convenient, and continuous glucose monitoring are feasible measures for preventing and treating diabetes-related cognitive dysfunction in the future. These measures will be of great clinical value and social benefits, especially for middle-aged and senior individuals with T2DM who are in relative danger of cognitive dysfunction.

Existing mechanism studies have partially revealed the underlying mechanisms of the association between hypoglycemia and cognitive dysfunction. It has been suggested that cognitive dysfunction after hypoglycemia is related to selective neuronal death, and its underlying mechanism involves excitotoxicity, oxidative stress, zinc release, PARP-1 activation, blood–brain barrier dysfunction and mitochondrial dysfunction (50–52). In addition, several recent studies have found that transient receptor potential canonical channel 6 (TRPC6) (53) and histidine metabolism (54) may be potential therapeutic targets for preventing cognitive impairment caused by hypoglycemia events, while verapamil (55), ketogenic diet (56), niacinamide mononucleotide (57), and pyruvate (58) may be effective measures for alleviating brain injury caused by hypoglycemia. However, the specific dose–response relationship and mechanism of hypoglycemic brain injury and its cumulative effect are still not fully understood, which limits its further guiding role in clinical practice. In the future, a more rigorous research must be more thorough and concentrate on the cumulative effect of hypoglycemic events, and the dose–response relationship and its pathological mechanism need to be further clarified through an in-depth research.

In recent years, multiple studies have focused on the link between hypoglycemic events and cognitive dysfunction risk, showing that the association between the two has received widespread attention. However, after an extensive literature search and careful literature review, we found that there were many deficiencies in the study design and report of results. For example, the diagnostic criteria of cognitive dysfunction was not completely unified, the severity, frequency and duration of hypoglycemia were not accurately quantified, and the specific types of cognitive dysfunction were not clearly distinguished. As a result, most studies produced relatively broad results and vague conclusions, which were difficult to solve relatively complex and specific clinical problems.

The complex nature of the connection between hypoglycemia and cognitive dysfunction cannot be overlooked, as it involves various confounding factors such as demographic characteristics, diabetes status, underlying diseases, medication, lifestyle, and genetics (59–61). Adjustments for confounders varied across the encompassed publication, which served as a substantial factor contributing to heterogeneity in this systematic review. At present, most studies rely on statistical correction methods to balance the confounding factors between the exposed group and the non-exposed group, which obviously cannot meet the needs of the current clinical research. Prospective studies with stratified block designs for confounding factors are urgently needed to obtain more rigorous results. Even though multiple research efforts have endeavored to investigate the independent risk variables of cognitive dysfunction in people with T2DM, such as duration, gender and education level (62), these specifics were not reported in depth in the findings report, which constrained the further subgroup analysis of this meta-analysis. To accurately prevent the onset and development of cognitive dysfunction in diabetic individuals who vary in duration, gender, and educational levels, more in-depth research should be encouraged to be conducted and reported in the future.

We must admit that this study still has the following shortcomings: (1) Despite the sensitivity analysis and identification of sources of heterogeneity, substantial heterogeneity remained among the included studies, such as the definition of cognitive dysfunction. (2) The study focused primarily on middle-aged and elderly individuals with T2DM, so there are potential limitations to generalizing the findings to other age groups or individuals with type 1 diabetes. (3) This study did not examine how cognitive dysfunction affected hypoglycemia episodes; it only focused on how hypoglycemic events affected the risk of developing cognitive dysfunction. The causal relationship between the two needs to be further studied. (4) Detailed characteristics of hypoglycemic events, such as the severity of hypoglycemia, the duration of hypoglycemia, the management of hypoglycemia, and specific categories of cognitive dysfunction were not specified in this study. (5) The effect of hypoglycemia events on cognitive function may have been amplified since the majority of the original study data included in this analysis came from hospital or medical claims records following severe hypoglycemic events.

## 5 Conclusion

In conclusion, the findings confirmed that hypoglycemia episodes may raise the possibility of cognitive dysfunction in those suffering from T2DM. It is recommended that pertinent departments concentrate on bolstering continuous glucose monitoring, routine cognitive screening, and moderate health education in middle-aged and seniors with T2DM to prevent diabetes-related cognitive dysfunction through efficient prevention and treatment of hypoglycemia events. This is in light of the current state of the treatment for cognition dysfunction and the findings of this study.

## Data Availability

The original contributions presented in the study are included in the article/Supplementary material, further inquiries can be directed to the corresponding authors.

## Supplementary material

SUPPLEMENTARY MATERIAL 1 Search strategy.

SUPPLEMENTARY MATERIAL 2 Characteristics of included studies.

SUPPLEMENTARY MATERIAL 3 Forest plot of the 16 trials.

SUPPLEMENTARY MATERIAL 4 Forest plot of the 14 trials.

## References

[1] ElSayed NA Aleppo G Aroda VR Bannuru RR Brown FM Bruemmer D . 2. Classification and diagnosis of Diabetes: standards of Care in Diabetes-2023. Diabetes Care. (2023) 46:S19–40. doi: 10.2337/dc23-S002, PMID: 36507649 PMC9810477

[2] Sun H Saeedi P Karuranga S Pinkepank M Ogurtsova K Duncan BB . IDF Diabetes atlas: global, regional and country-level diabetes prevalence estimates for 2021 and projections for 2045. Diabetes Res Clin Pract. (2022) 183:109119. doi: 10.1016/j.diabres.2021.109119, PMID: 34879977 PMC11057359

[3] Zheng Y Ley SH Hu FB. Global aetiology and epidemiology of type 2 diabetes mellitus and its complications. Nat Rev Endocrinol. (2018) 14:88–98. doi: 10.1038/nrendo.2017.151 29219149

[4] Biessels GJ Despa F. Cognitive decline and dementia in diabetes mellitus: mechanisms and clinical implications. Nat Rev Endocrinol. (2018) 14:591–604. doi: 10.1038/s41574-018-0048-7, PMID: 30022099 PMC6397437

[5] Dove A Shang Y Xu W Grande G Laukka EJ Fratiglioni L . The impact of diabetes on cognitive impairment and its progression to dementia. Alzheimers Dement. (2021) 17:1769–78. doi: 10.1002/alz.12482 34636485

[6] Ciudin A Espinosa A Simó-Servat O Ruiz A Alegret M Hernández C . Type 2 diabetes is an independent risk factor for dementia conversion in patients with mild cognitive impairment. J Diabetes Complicat. (2017) 31:1272–4. doi: 10.1016/j.jdiacomp.2017.04.018, PMID: 28545893

[7] Chen X Li W Huang Y Yang J Tao Y Huang L . Association of Type 2 Diabetes mellitus with cognitive function in adults: a prospective cohort study. J Alzheimers Dis. (2023) 93:1509–20. doi: 10.3233/jad-220822 37212092

[8] Honig LS Vellas B Woodward M Boada M Bullock R Borrie M . Trial of Solanezumab for mild dementia due to Alzheimer's disease. N Engl J Med. (2018) 378:321–30. doi: 10.1056/NEJMoa1705971, PMID: 29365294

[9] Ostrowitzki S Lasser RA Dorflinger E Scheltens P Barkhof F Nikolcheva T . A phase III randomized trial of gantenerumab in prodromal Alzheimer's disease. Alzheimers Res Ther. (2017) 9:95. doi: 10.1186/s13195-017-0318-y, PMID: 29221491 PMC5723032

[10] Battaglia S Avenanti A Vécsei L Tanaka M. Neural correlates and molecular mechanisms of memory and learning. Int J Mol Sci. (2024) 25:2724. doi: 10.3390/ijms25052724, PMID: 38473973 PMC10931689

[11] Battaglia S Di Fazio C Mazzà M Tamietto M Avenanti A. Targeting human glucocorticoid receptors in fear learning: a multiscale integrated approach to study functional connectivity. Int J Mol Sci. (2024) 25:864. doi: 10.3390/ijms25020864, PMID: 38255937 PMC10815285

[12] Cho JH Suh S. Glucocorticoid-induced hyperglycemia: a neglected problem. Endocrinol Metab. (2024) 39:222–38. doi: 10.3803/EnM.2024.1951, PMID: 38532282 PMC11066448

[13] Kho AR Choi BY Lee SH Hong DK Kang BS Lee SH . Administration of an Acidic Sphingomyelinase (ASMase) inhibitor, imipramine, reduces hypoglycemia-induced hippocampal neuronal death. Cells. (2022) 11:667. doi: 10.3390/cells11040667, PMID: 35203316 PMC8869983

[14] Lin L Chen Z Huang C Wu Y Huang L Wang L . Mito-TEMPO, a mitochondria-targeted antioxidant, improves cognitive dysfunction due to hypoglycemia: an Association with reduced Pericyte loss and blood-brain barrier leakage. Mol Neurobiol. (2023) 60:672–86. doi: 10.1007/s12035-022-03101-0, PMID: 36357613

[15] Zhou Y Huang L Zheng W An J Zhan Z Wang L . Recurrent nonsevere hypoglycemia exacerbates imbalance of mitochondrial homeostasis leading to synapse injury and cognitive deficit in diabetes. Am J Physiol Endocrinol Metab. (2018) 315:E973–e986. doi: 10.1152/ajpendo.00133.2018, PMID: 29969317

[16] He C Li Q Cui Y Gao P Shu W Zhou Q . Recurrent moderate hypoglycemia accelerates the progression of Alzheimer's disease through impairment of the TRPC6/GLUT3 pathway. JCI Insight. (2022) 7:e154595. doi: 10.1172/jci.insight.154595 35077394 PMC8983129

[17] Alkabbani W Maxwell CJ Marrie RA Tyas SL Lega IC Gamble JM. Associations of mid-and late-life severe hypoglycemic episodes with incident dementia among patients with type 2 Diabetes: a population-based cohort study. Diabetes Care. (2023) 46:331–40. doi: 10.2337/dc22-1496, PMID: 36516080

[18] Li CY Kuo CL Chang YH Lu CL Martini S Hou WH. Association between trajectory of severe hypoglycemia and dementia in patients with type 2 Diabetes: a population-based study. J Epidemiol. (2022) 32:423–30. doi: 10.2188/jea.JE20200518, PMID: 33678721 PMC9359896

[19] Han E Han KD Lee BW Kang ES Cha BS Ko SH . Severe hypoglycemia increases dementia risk and related mortality: a Nationwide, population-based cohort study. J Clin Endocrinol Metab. (2022) 107:e1976–86. doi: 10.1210/clinem/dgab860, PMID: 35015886

[20] Kim YG Park DG Moon SY Jeon JY Kim HJ Kim DJ . Hypoglycemia and dementia risk in older patients with type 2 Diabetes mellitus: a propensity-score matched analysis of a population-based cohort study. Diabetes Metab J. (2020) 44:125–33. doi: 10.4093/dmj.2018.0260, PMID: 31701690 PMC7043983

[21] Cukierman-Yaffe T Bosch J Jung H Punthakee Z Gerstein HC. Hypoglycemia and incident cognitive dysfunction: a post hoc analysis from the ORIGIN trial. Diabetes Care. (2019) 42:142–7. doi: 10.2337/dc18-0690 30425095

[22] Jacobson AM Musen G Ryan CM Silvers N Cleary P Waberski B . Long-term effect of diabetes and its treatment on cognitive function. N Engl J Med. (2007) 356:1842–52. doi: 10.1056/NEJMoa066397, PMID: 17476010 PMC2701294

[23] Bruce DG Davis WA Casey GP Clarnette RM Brown SG Jacobs IG . Severe hypoglycaemia and cognitive impairment in older patients with diabetes: the Fremantle Diabetes study. Diabetologia. (2009) 52:1808–15. doi: 10.1007/s00125-009-1437-1, PMID: 19575177

[24] Gómez-Guijarro MD Álvarez-Bueno C Saz-Lara A Sequí-Domínguez I Lucerón-Lucas-Torres M Cavero-Redondo I. Association between severe hypoglycaemia and risk of dementia in patients with type 2 diabetes mellitus: a systematic review and meta-analysis. Diabetes Metab Res Rev. (2023) 39:e3610. doi: 10.1002/dmrr.3610, PMID: 36649373

[25] Ye M Yuan AH Yang QQ Li QW Li FY Wei Y. Association of hypoglycemic events with cognitive impairment in patients with type 2 diabetes mellitus: protocol for a dose-response meta-analysis. PLoS One. (2024) 19:e0296662. doi: 10.1371/journal.pone.0296662, PMID: 38306364 PMC10836671

[26] Cumpston MS McKenzie JE Welch VA Brennan SE. Strengthening systematic reviews in public health: guidance in the Cochrane handbook for systematic reviews of interventions, 2nd edition. J Public Health (Oxf). (2022) 44:e588–92. doi: 10.1093/pubmed/fdac036, PMID: 35352103 PMC9715291

[27] Page MJ McKenzie JE Bossuyt PM Boutron I Hoffmann TC Mulrow CD . The PRISMA 2020 statement: an updated guideline for reporting systematic reviews. Syst Rev. (2021) 10:89. doi: 10.1186/s13643-021-01626-4, PMID: 33781348 PMC8008539

[28] Stroup DF Berlin JA Morton SC Olkin I Williamson GD Rennie D . Meta-analysis of observational studies in epidemiology: a proposal for reporting. Meta-analysis of observational studies in epidemiology (MOOSE) group. JAMA. (2000) 283:2008–12. doi: 10.1001/jama.283.15.2008, PMID: 10789670

[29] Amiel SA. The consequences of hypoglycaemia. Diabetologia. (2021) 64:963–70. doi: 10.1007/s00125-020-05366-3, PMID: 33550443 PMC8012317

[30] Wells G Shea B O Connell D Peterson J Welch V Losos M . The Newcastle-Ottawa Scale (NOS) for assessing the quality of nonrandomised studies in meta-analyses [EB/OL]. (2022). Available at: http://wwwohrica/programs/clinical_epidemiology/oxfordasp (Accessed July 2, 2023).

[31] DerSimonian R Kacker R. Random-effects model for meta-analysis of clinical trials: an update. Contemp Clin Trials. (2007) 28:105–14. doi: 10.1016/j.cct.2006.04.004 16807131

[32] Iso-Markku P Kujala UM Knittle K Polet J Vuoksimaa E Waller K. Physical activity as a protective factor for dementia and Alzheimer's disease: systematic review, meta-analysis and quality assessment of cohort and case-control studies. Br J Sports Med. (2022) 56:701–9. doi: 10.1136/bjsports-2021-104981, PMID: 35301183 PMC9163715

[33] VanderWeele TJ. On a square-root transformation of the odds ratio for a common outcome. Epidemiology. (2017) 28:e58–60. doi: 10.1097/ede.0000000000000733, PMID: 28816709 PMC5617805

[34] Zhang J Yu KF. What's the relative risk? A method of correcting the odds ratio in cohort studies of common outcomes. JAMA. (1998) 280:1690–1. doi: 10.1001/jama.280.19.1690 9832001

[35] Higgins JP Thompson SG. Quantifying heterogeneity in a meta-analysis. Stat Med. (2002) 21:1539–58. doi: 10.1002/sim.1186, PMID: 12111919

[36] Sterne JA Egger M Smith GD. Systematic reviews in health care: investigating and dealing with publication and other biases in meta-analysis. BMJ. (2001) 323:101–5. doi: 10.1136/bmj.323.7304.101 11451790 PMC1120714

[37] Hou X Wang C Du R. The status of cognitive function and its influencing factors in elderly patients with diabetes mellitus. Chin J Public Health Eng. (2019) 18:543–5. doi: 10.19937/j.issn.1671-4199.2019.04.022

[38] Liu C Li M. Influencing factors of cognitive impairment in patients with type 2 diabetes mellitus. Chin J Clinic Res. (2016) 29:1362–5. doi: 10.13429/j.cnki.cjcr.2016.10.020

[39] Mao H. The cognitive function level and related influencing factors in elderly patients with type 2 diabetes mellitus. Prac J Clinic Med. (2019) 16:114–7.

[40] Zhang J Wang Q Cao H. Correlation between hypoglycemia and cognitive dysfunction in elderly diabetes mellitus. Chin J Gen Prac. (2021) 19:1677–9. doi: 10.16766/j.cnki.issn.1674-4152.002140

[41] Chin SO Rhee SY Chon S Baik SH Park Y Nam MS . Hypoglycemia is associated with dementia in elderly patients with type 2 diabetes mellitus: An analysis based on the Korea National Diabetes Program Cohort. Diabetes Res Clin Pract. (2016) 122:54–61. doi: 10.1016/j.diabres.2016.09.027, PMID: 27810686

[42] Feinkohl I Aung PP Keller M Robertson CM Morling JR McLachlan S . Severe hypoglycemia and cognitive decline in older people with type 2 diabetes: the Edinburgh type 2 diabetes study. Diabetes Care. (2014) 37:507–15. doi: 10.2337/dc13-1384, PMID: 24103900

[43] Haroon NN Austin PC Shah BR Wu J Gill SS Booth GL. Risk of dementia in seniors with newly diagnosed diabetes: a population-based study. Diabetes Care. (2015) 38:1868–75. doi: 10.2337/dc15-0491 26216873

[44] Lin CH Sheu WH. Hypoglycaemic episodes and risk of dementia in diabetes mellitus: 7-year follow-up study. J Intern Med. (2013) 273:102–10. doi: 10.1111/joim.12000, PMID: 23003116

[45] Liu S Lu Y Cai X Cong R Li J Jiang H . Glycemic control is related to cognitive dysfunction in elderly people with type 2 Diabetes mellitus in a rural Chinese population. Curr Alzheimer Res. (2019) 16:950–62. doi: 10.2174/1567205016666191023110712, PMID: 31642779

[46] Mehta HB Mehta V Goodwin JS. Association of Hypoglycemia with Subsequent Dementia in older patients with type 2 Diabetes mellitus. J Gerontol A Biol Sci Med Sci. (2017) 72:glw217–glw1116. doi: 10.1093/gerona/glw217, PMID: 27784724 PMC5861972

[47] Whitmer RA Karter AJ Yaffe K Quesenberry CP Jr Selby JV. Hypoglycemic episodes and risk of dementia in older patients with type 2 diabetes mellitus. JAMA. (2009) 301:1565–72. doi: 10.1001/jama.2009.460, PMID: 19366776 PMC2782622

[48] Yaffe K Falvey CM Hamilton N Harris TB Simonsick EM Strotmeyer ES . Association between hypoglycemia and dementia in a biracial cohort of older adults with diabetes mellitus. JAMA Intern Med. (2013) 173:1300–6. doi: 10.1001/jamainternmed.2013.6176, PMID: 23753199 PMC4041621

[49] Shen X Dong Y Wang L Gong C Gao P. Relationship between plasma tau protein, ptau protein level and cognitive function in elderly patients with hypoglycemia. Chin J Diabetes. (2018) 26:559–62.

[50] Languren G Montiel T Julio-Amilpas A Massieu L. Neuronal damage and cognitive impairment associated with hypoglycemia: An integrated view. Neurochem Int. (2013) 63:331–43. doi: 10.1016/j.neuint.2013.06.018, PMID: 23876631

[51] Lin L Wu Y Chen Z Huang L Wang L Liu L. Severe hypoglycemia contributing to cognitive dysfunction in diabetic mice is associated with Pericyte and blood-brain barrier dysfunction. Front Aging Neurosci. (2021) 13:775244. doi: 10.3389/fnagi.2021.775244, PMID: 34899278 PMC8662820

[52] Wu K Huang C Zheng W Wu Y Huang Q Lin M . Activation of mitophagy improves cognitive dysfunction in diabetic mice with recurrent non-severe hypoglycemia. Mol Cell Endocrinol. (2024) 580:112109. doi: 10.1016/j.mce.2023.112109, PMID: 37956789

[53] He C Gao P Cui Y Li Q Li Y Lu Z . Low-glucose-sensitive TRPC6 dysfunction drives hypoglycemia-induced cognitive impairment in diabetes. Clin Transl Med. (2020) 10:e205. doi: 10.1002/ctm2.205, PMID: 33135341 PMC7568851

[54] Wu K Xie W Chen Z Zhou L Wang L Zhou Y . Disturbed hippocampal histidine metabolism contributes to cognitive impairment induced by recurrent nonsevere hypoglycemia in diabetes. Biochem Biophys Res Commun. (2023) 682:325–34. doi: 10.1016/j.bbrc.2023.10.036, PMID: 37837753

[55] Jackson DA Michael T Vieira de Abreu A Agrawal R Bortolato M Fisher SJ. Prevention of severe hypoglycemia-induced brain damage and cognitive impairment with verapamil. Diabetes. (2018) 67:2107–12. doi: 10.2337/db18-0008, PMID: 29724724 PMC6152340

[56] Li C Ma Y Chai X Feng X Feng W Zhao Y . Ketogenic diet attenuates cognitive dysfunctions induced by hypoglycemia via inhibiting endoplasmic reticulum stress-dependent pathways. Food Funct. (2024) 15:1294–309. doi: 10.1039/d3fo04007k, PMID: 38197246

[57] Wang X Hu X Zhang L Xu X Sakurai T. Nicotinamide mononucleotide administration after sever hypoglycemia improves neuronal survival and cognitive function in rats. Brain Res Bull. (2020) 160:98–106. doi: 10.1016/j.brainresbull.2020.04.022, PMID: 32380185

[58] Suh SW Aoyama K Matsumori Y Liu J Swanson RA. Pyruvate administered after severe hypoglycemia reduces neuronal death and cognitive impairment. Diabetes. (2005) 54:1452–8. doi: 10.2337/diabetes.54.5.1452, PMID: 15855333

[59] Shih IF Paul K Haan M Yu Y Ritz B. Physical activity modifies the influence of apolipoprotein E ε4 allele and type 2 diabetes on dementia and cognitive impairment among older Mexican Americans. Alzheimers Dement. (2018) 14:1–9. doi: 10.1016/j.jalz.2017.05.005, PMID: 28692819 PMC5750101

[60] Liu J Yang W Luo H Ma Y Zhao H Dan X. Brain-derived neurotrophic factor Val66Met polymorphism is associated with mild cognitive impairment in elderly patients with type 2 diabetes: a case-controlled study. Aging Clin Exp Res. (2021) 33:1659–66. doi: 10.1007/s40520-020-01687-w, PMID: 32892314

[61] Tong XW Zhang YT Yu ZW Pu SD Li X Xu YX . Triglyceride glucose index is related with the risk of mild cognitive impairment in type 2 Diabetes. Diabetes Metab Syndr Obes. (2022) 15:3577–87. doi: 10.2147/dmso.S389327, PMID: 36426213 PMC9680968

[62] Sun L Diao X Gang X Lv Y Zhao X Yang S . Risk factors for cognitive impairment in patients with type 2 Diabetes. J Diabetes Res. (2020) 2020:4591938–10. doi: 10.1155/2020/4591938, PMID: 32377520 PMC7196145

