# Supplementary figures and images for: Effect of hypoglycemic events on cognitive function in individuals with type 2 diabetes mellitus: a dose–response meta-analysis
Source: Front Neurol. 2024 Aug 13;15:1394499. doi: 10.3389/fneur.2024.1394499 (PMC11347434; doi:10.3389/fneur.2024.1394499)

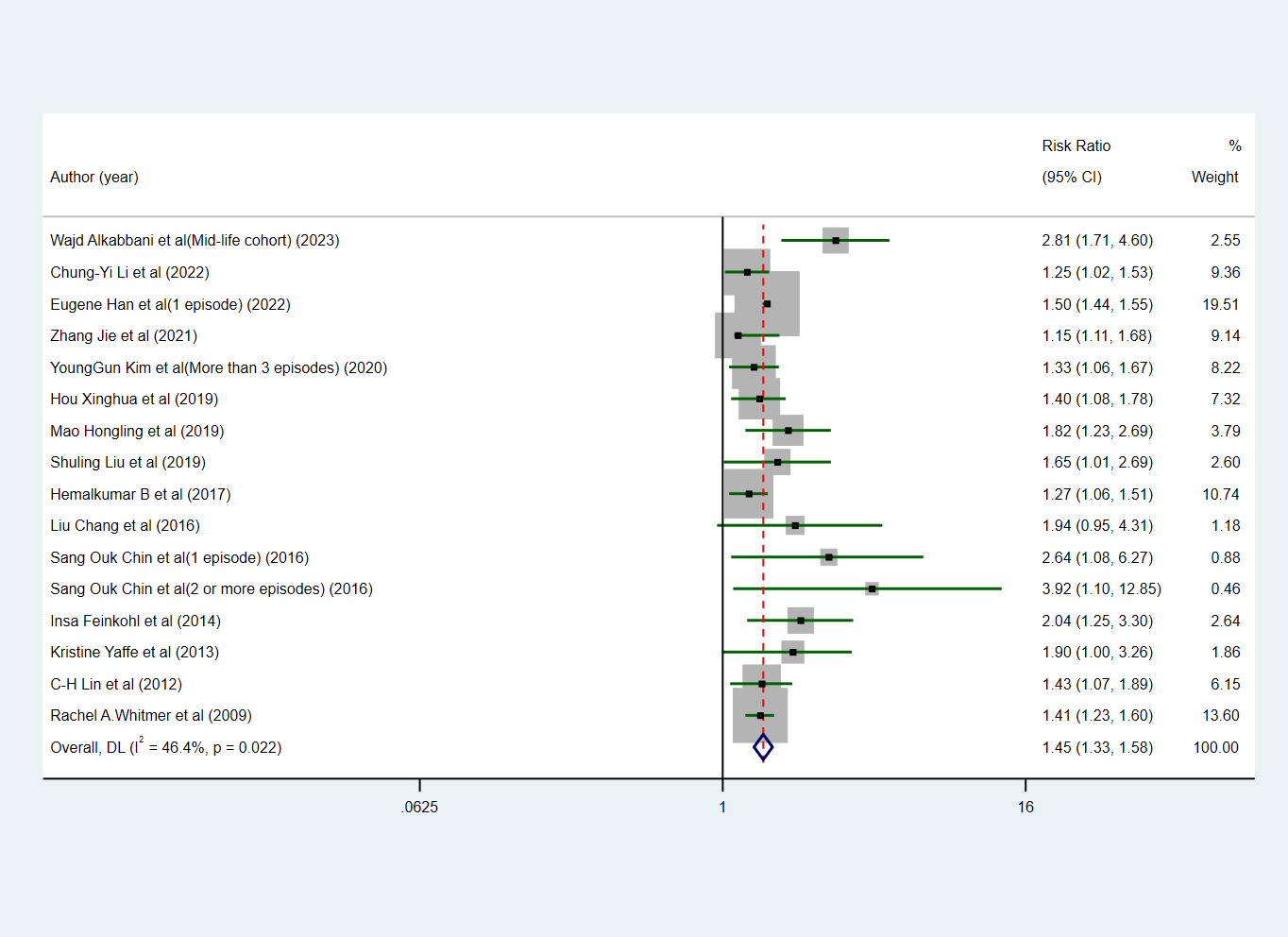

Supplement: SUPPLEMENTARY MATERIAL 1 — Search strategy. [file Image_1.tif]

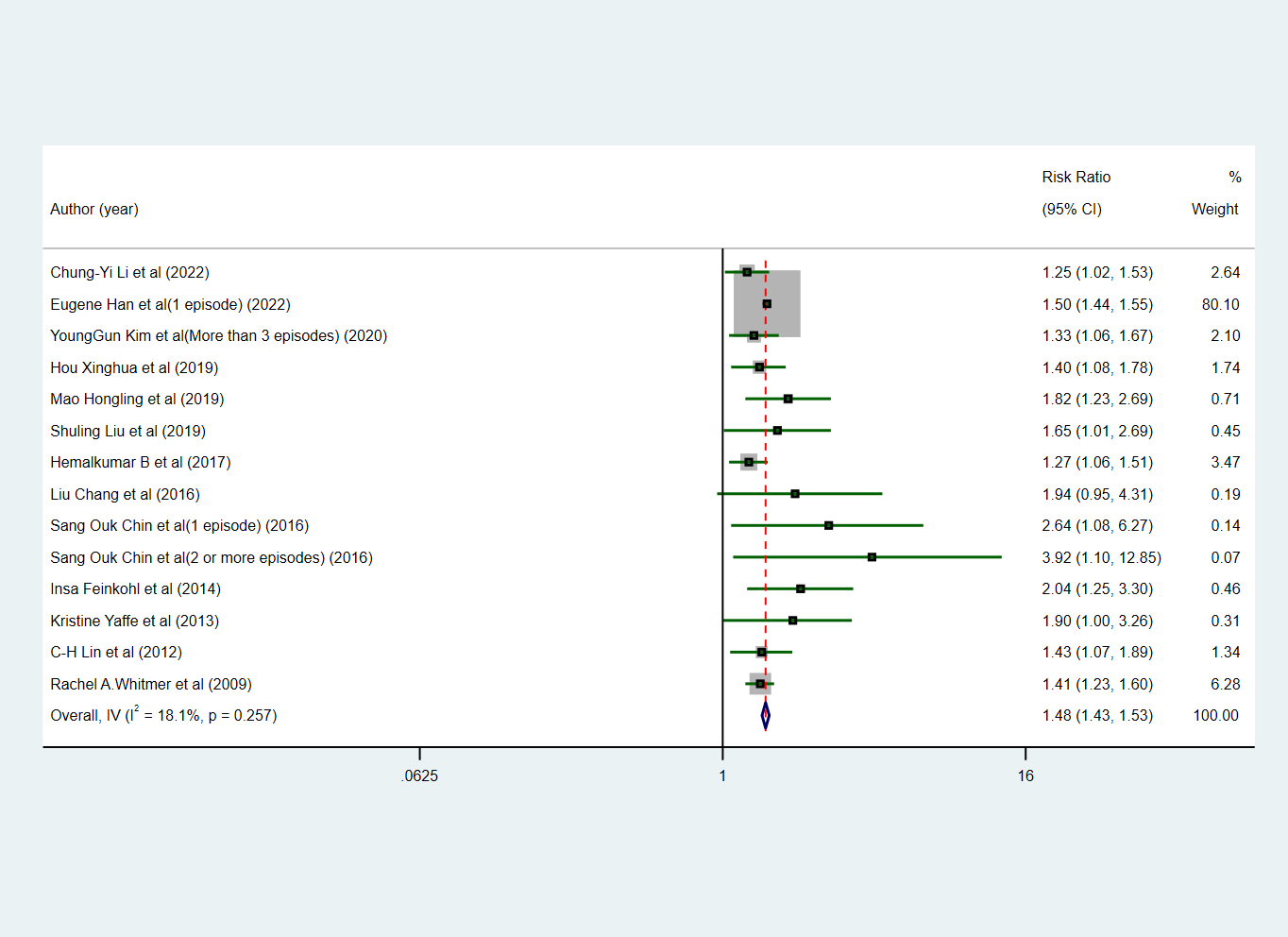

Supplement: SUPPLEMENTARY MATERIAL 2 — Characteristics of included studies. [file Image_2.tif]
