# Supplementary material for: Effect of hypoglycemic events on cognitive function in individuals with type 2 diabetes mellitus: a dose–response meta-analysis
Source: Front Neurol. 2024 Aug 13;15:1394499. doi: 10.3389/fneur.2024.1394499 (PMC11347434; doi:10.3389/fneur.2024.1394499)
Supplement: SUPPLEMENTARY MATERIAL 3 — Forest plot of the 16 trials. [file Table_1.docx]

**Search strategy**

**Take PubMed as an example**

**#1 ((Diabetes Mellitus,Type 2[MeSH Terms]) OR (noninsulin[Title/Abstract])) OR (noninsulin-dependent Diabetes Mellitus[Title/Abstract])) OR (Type 2 diabetes[Title/Abstract])) OR (Type 2 diabetes Mellitus[Title/Abstract])) OR (Type 2 diabetic[Title/Abstract])) OR (T2DM[Title/Abstract])) OR (DM[Title/Abstract]))**

**#2 (((Cognitive Dysfunction[MeSH Terms]) OR (Cognition Disorders[Title/Abstract])) OR (Cognitive Disorder[Title/Abstract])) OR (Dementia[Title/Abstract])) OR (cognitive decline[Title/Abstract])) OR (cognition disorder[Title/Abstract])) OR (cognition disorder[Title/Abstract])) OR (cognitive impairment[Title/Abstract])) OR (executive function[Title/Abstract])) OR (cognitive function[Title/Abstract])) OR (memory[Title/Abstract])))**

**#3 ((Risk factor[Title/Abstract]) OR (predicted[Title/Abstract])) OR (predictor[Title/Abstract])) OR (risk[Title/Abstract])) OR (relat[Title/Abstract])) OR (associat[Title/Abstract])) OR (factor[Title/Abstract])) OR (reason[Title/Abstract])) OR (correlated[Title/Abstract])) OR (predictor[Title/Abstract])) OR (influen[Title/Abstract])) OR (inciden[Title/Abstract]))**

**#4 #1 AND #2 AND #3**
